# *Mycobacterium xenopi* Laryngitis: A Case Report of an Unusual Presentation and Diagnostic Challenge

**DOI:** 10.1155/crdi/9725815

**Published:** 2025-12-02

**Authors:** Kelly E. Daniels, Shaidy Moronta, Jacob Kaufman, Mohamed Yassin, Libby J. Smith

**Affiliations:** ^1^Department of Otolaryngology Head and Neck Surgery, University of Pittsburgh Medical Center, Pittsburgh, Pennsylvania, USA; ^2^Department of General Surgery, Danbury Hospital, Danbury, Connecticut, USA; ^3^Department of Orthopedic Surgery, University of Pittsburgh Medical Center, Pittsburgh, Pennsylvania, USA; ^4^Department of Infectious Diseases, University of Pittsburgh Medical Center, Pittsburgh, Pennsylvania, USA

## Abstract

*Mycobacterium xenopi* (*M. xenopi)* is a less common nontuberculous mycobacterium (NTM) responsible for pulmonary and other infections and can be a diagnostic and therapeutic challenge requiring prolonged courses of triple-drug therapy. We managed a case of isolated *M. xenopi* laryngitis in an immunocompetent patient after they presented with dysphonia and a nonspecific unilateral vocal fold lesion. This patient was treated with complete surgical excision alone, and negative cultures and symptom resolution were achieved in the absence of antimicrobials. Laryngeal infection by *M. xenopi* is a rare diagnosis, so it is important to keep NTM infections on the differential, and once confirmed by pathology and culture, to be aware of the option for surgical excision for definitive treatment.

## 1. Introduction

*Mycobacterium xenopi* (*M. xenopi)* is a less common nontuberculous mycobacterium (NTM) often affecting the lungs. *M. xenopi,* like other mycobacteria, can cause infection or colonization, and differentiation between the two states can be a clinical challenge. Additionally, there is limited data about *M. xenopi* therapeutic options. This is driven by challenging susceptibility testing, and due to the low incidence of infections, insufficient evidence to make standardized treatment guidelines [1, 2]. The guidelines for treatment of NTM pulmonary disease recommend at least 12 months of treatment with a three-drug regimen, though the exact regimen remains up to the individual clinician, patient, and relevant risks and benefits given the clinical scenario. The in vitro data suggest the use of a macrolide and a fluoroquinolone (both for at least 12 months), along with intravenous amikacin (typically for 8–12 weeks) whereas the most recent clinical practice guideline from the Infectious Diseases Society of America offers a “conditional recommendation” for a combination of rifampicin, ethambutol, and either a macrolide and/or a fluoroquinolone [3]. *M. xenopi* is associated with increased mortality, and antimicrobial therapy is recommended despite the paucity of data on antimicrobial susceptibility and efficacy [3].

Literature on *M. xenopi* infections is largely restricted to case reports, a majority of which describe pulmonary infections, with no reported instances of *M. xenopi*-associated laryngitis. We report a case of successfully eradicated laryngeal *M. xenopi* which we believe is the first in the literature. No written consent has been obtained from the patients as there is no patient identifiable data included in this case report.

## 2. Case Presentation

**Figure 1:**
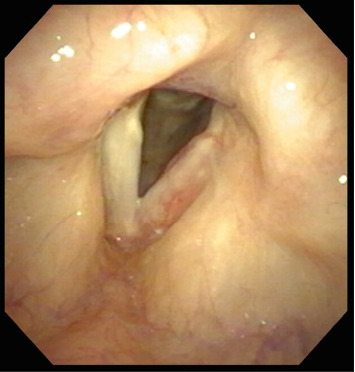
Flexible laryngoscopy showing hyperemic, edematous left true vocal fold with white discoloration and adynamic mucosal wave. The right true vocal fold is normal. There was normal bilateral vocal fold motion.

A 77-year-old female nonsmoker with a history of hypertension, asthma, and reflux presented to her primary care provider with acute-onset dysphonia for one month duration. She was not on inhaled steroids at baseline. She was empirically prescribed Duonebs and a prednisone taper without improvement. When she presented to the general otolaryngology clinic 3 months later, she described her voice as raspy and strained, at 60% of normal. Flexible laryngoscopy demonstrated a thickened, erythematous left true vocal fold with an irregular white plaque. She completed another 10-day prednisone taper and was referred to our tertiary voice center. Flexible videostroboscopy confirmed the presence of an unchanged hypervascular, polypoid lesion of the left true vocal fold, with an absent mucosal wave. Vocal fold adduction and abduction were preserved (Figure 1).

**Figure 2:**
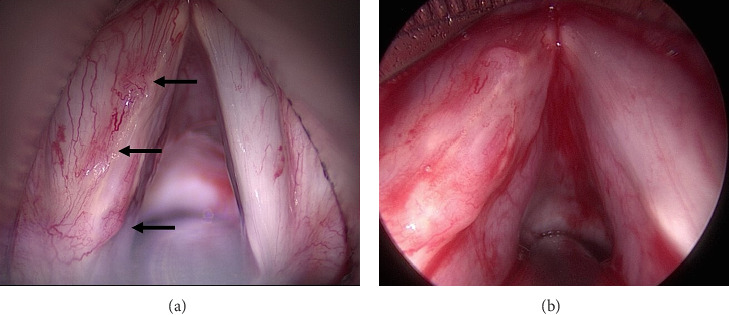
Intraoperative examination, showing hypervascularity, polypoid consolidations of raised epithelial tissue of left true vocal fold. (a) View using 0° rigid endoscope. (b) View using 30° rigid endoscope.

She underwent microsuspension laryngoscopy with microflap excision of the left true vocal fold lesion (Figure 2). Acid-fast bacilli cultures (AFB) were positive for *Mycobacterium xenopi* and histology demonstrated multiple noncaseating granulomas.

**Figure 3:**
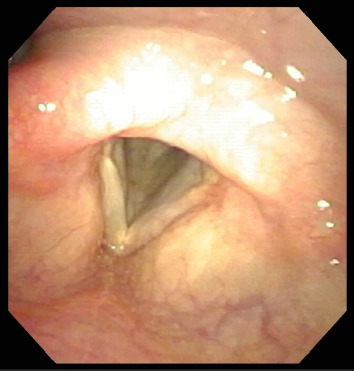
Flexible laryngoscopy, 12 months postoperatively, showing resolution of left vocal fold hyperemia with no residual varicosities. Mucosal wave improved with moderate residual scarring noted on stroboscopy.

The infectious diseases team recommended neck and chest computed tomography to identify a primary source, which was unremarkable. Subsequent AFB cultures were ordered to monitor infection; however, all postoperative cultures remained negative, suggesting that surgical excision was therapeutic. She did not require any antimycobacterial medical therapy. She was monitored with interval laryngoscopy, and 12 months after surgery, she continued to be asymptomatic with normal bilateral vocal fold motion and no evidence of infection (Figure 3). She reported complete restoration of normal voice quality.

We reviewed electronic health records for cases of NTM in our hospital system in Western Pennsylvania between Jan. 2016 and Dec. 2023 and identified 1253 patients who had at least one NTM isolated, as either infection or colonization, summarized in Table 1.

## 3. Discussion

This is the first case report of *M. xenopi* laryngitis to be reported in the literature. This patient was treated successfully with surgical source control and no additional mycobacterial antibiotic therapy. Endoscopy findings were nonspecific for mycobacterial laryngeal infection, and both diagnosis and treatment were made via surgical excision. In general, there is a broad differential for nonspecific vocal fold lesions which includes infectious, inflammatory, and rheumatologic etiologies as well as benign and malignant lesions alike. A diagnosis is often ultimately made based on histology. As such, for atypical lesions, the authors recommend sending biopsy specimens for both histology and culture, including dedicated AFB staining. Our patient was neither immunocompromised nor a smoker. We did not identify any other risk factors for *M. xenopi* infection other than a remote history of travel to Vietnam.

Cases of other NTM involving the larynx are reported in the literature, all of which were treated with a combination of surgery and medical therapy. A case of *Mycobacterium kansasii* is presented by Lehman et al. in a 58-year-old female with an extensive travel history throughout the developing world, who developed laryngitis while living in West Africa [4]. She remained symptomatic for 5 years and was ultimately diagnosed with NTM after bilateral microflaps were used to excise bilateral inflammatory vocal fold lesions. She underwent triple-drug therapy (isoniazid, rifampin, and ethambutol) for 6 months with resultant symptomatic improvement. Lau et al. presented a 68-year-old female former smoker with concurrent pulmonary disease who presented with vocal fold ulceration and an exophytic tissue mass in the subglottis and trachea, subsequently diagnosed with *Mycobacterium abscessus* infection [5]. The mass was excised by wedge resection via carbon dioxide laser ablation and two months of IV triple-drug therapy (cefoxitin, imipenem, and amikacin) followed by 24 months of oral triple-drug therapy (azithromycin, clofazimine, and inhaled amikacin). She developed subglottic stenosis which required further surgical intervention and another 8 months of antibiotic therapy, though ultimately, she was able to achieve a stable airway and negative cultures.

The above cases both present patients with one or more identifiable risk factors for NTM; in contrast, our patient appeared to be without a risk factor. McEwan et al. similarly presented a case from a 66-year-old healthy female who was diagnosed with *Mycobacterium malmoense* involving her right vocal fold that resolved with 12 months of five-drug therapy (rifampicin, isoniazid, ethambutol, pyridoxine, and clarithromycin) [6].

While each of the prior cases utilized multidrug therapy, our case demonstrates that surgical ablation with clear margins may supplant the need for prolonged antimicrobial therapy in certain circumstances. Support for surgical eradication of NTM infections is strongest in the pediatric literature for NTM cervicofacial lymphadenitis, where according to the most recent consensus guidelines complete surgical excision is favored when risk to the facial nerve or other important structures is minimized [7]. When considering therapy for laryngeal NTM, the clinician should consider whether excision would be possible while preserving an acceptable vocal quality as well as ease of serial cultures, reliability of patient follow-up, and risks of delaying therapy. As we report here on the successful surgical eradication specifically of *M. xenopi,* more data would be needed to generalize to other species of NTM.

## 4. Conclusion

Laryngeal infection by *M. xenopi* is a rare clinical scenario that can affect immunocompetent patients. In cases of abnormal laryngeal lesions, NTM infection should remain on the differential, and biopsies should be sent for pathology and culture, including aerobe, anaerobe, fungal, and AFB assessment. For focal laryngeal *M. xenopi,* a complete surgical excision may be sufficient to eradicate disease, negating the need for long-term systemic antibiotics.

## Figures and Tables

**Table 1:** Regional characterization of nontuberculous mycobacterial infection from January 2016 through December 2023.

Nontuberculous mycobacterial cohort	No. (%) of patients
Total	1253
Male	629 (50)
Female	624 (50)
Average age (range)	68 (8–97)
*M. xenopi* cases cohort	
Total no. Isolated	12
Male	9 (75)
Female	3 (25)
Average age (range)	65 (48–85)
Immunocompromised	10 (83)
Colonized/contaminant	6 (50)
True infection	6 (50)
Treatment for true infections:	
Systemic antibiotic therapy	5 (83)
Surgery without antibiotics^∗^	1 (17)

^∗^Our patient.

## Data Availability

The data that support the findings of this study are available from the corresponding author upon reasonable request.
